# Effects of the ninein-like protein centrosomal protein on breast cancer cell invasion and migration

**DOI:** 10.3892/mmr.2015.3650

**Published:** 2015-04-20

**Authors:** QI LIU, XINZHAO WANG, MINLIN LV, DIANBIN MU, LEILEI WANG, WENSU ZUO, ZHIYONG YU

**Affiliations:** 1 Department of II, Shandong Breast Center of Prevention and Treatment, Shandong Cancer Hospital, Jinan, Shandong 250117, P.R. China; 2 Clinical Laboratory, Haiyang People’s Hospital, Yantai, Shandong 265100, P.R. China

**Keywords:** breast cancer, centrosomal protein, ninein-like protein, tumor invasion, tumor metastasis

## Abstract

To investigate the effects of the centrosomal protein, ninein-like protein (Nlp), on the proliferation, invasion and metastasis of MCF-7 breast cancer cells, the present study established green fluorescent protein (GFP)-containing MCF7 plasmids with steady and overexpression of Nlp (MCG7-GFP-N1p) and blank plasmids (MCF7-GFP) using lentiviral transfection technology in MCF7 the breast cancer cell line. The expression of Nlp was determined by reverse transcription-quantitative polymerase chain reaction and western blott analysis. Differences in levels of proliferation, invasion and metastasis between the MCF7-GFP-Nlp group and MCF-GFP group were compared using MTT, plate colony formation and Transwell migration assays. The cell growth was more rapid and the colony forming rate was markedly increased in the MCF7-GFP-Nlp group (P<0.05) compared with the MCF7-GFP group. The number of cells in the MCF-GFP-Nlp and MCF7-GFP groups transferred across membranes were 878±18.22 and 398±8.02, respectively, in the migration assay. The invasive capacity was significantly increased in the MCF7-GFP-Nlp group (P<0.05) compared with the MCF7-GFP group. The western blotting results demonstrated high expression levels of C-X-C chemokine receptor type 4 in the MCF7-GFP-Nlp group. The increased expression of Nlp was associated with an increase in MCF7 cell proliferation, invasion and metastasis, which indicated that Nlp promoted breast tumorigenesis and may be used as a potent biological index to predict breast cancer metastasis and develop therapeutic regimes.

## Introduction

Breast cancer is a malignant tumor, which severely affects female health, is life threatening and the incidence of which has increased gradually over recent years (1,2). With the prevalence of advanced diagnostic instruments and the development of standardized systematic therapy, the rate of early diagnosis in patients with recurrent-metastasis patients has increased and survival rates have improved, with mortality rates declining by 1–2% per year in China (1,2). The present study aimed to investigate the risk factors of breast cancer, recurrent-metastasis and intervention methods, which are important to decrease the breast cancer mortality rate. The human centrosomal ninein-like protein (Nlp) is a novel member of the γ-tubulin complex binding proteins (GTBPs) and is essential in the process of mitosis. The primary function of Nlp is to promote microtubule nucleation, which contributes to centrosomal maturation, spindle formation and chromosome segregation (3,4). The centrosome from almost all types of tumor exhibit abnormal structure, morphology and function. Previous studies have demonstrated that centrosome activity is important in cell division and in the transition from G1 phase to S phase (5,6). Abnormal centrosomes may lead to interruption of the cell cycle, including the polycaryon phenotype, which causes abnormal cell transformation, tumorigenesis and the development of malignancy (7–9). In the present study, the biological action of Nlp on metastatic capacity of breast cancer was investigated using advanced transfection technology.

## Materials and methods

### Cell culture

The MCF-7 breast cancer cell line was provided by the Basic Center of Shandong Tumor Hospital (Shandong, China), and the cells were cultured in Dulbecco’s modified Eagle’s medium (DMEM; Hyclone Corporation, Logan, USA), containing 10% fetal bovine serum (Hyclone Corporation), 100 U/ml penicillin and 100 pg/ml streptomycin (Beyotime Institute of Biotechnology, Haimen, China) in a humidified incubator at 37°C under 5% CO_2_.

### Steady transfection with lentivirus

The MCF-7 cells were seeded into 10 cm cell culture bottles (6×10^5^ cells/ml) containing 10% serum and medium 24 h prior to transfection and were cultured in a humidified incubator at 37°C under 5% CO_2_. The cells were not transfected until the cell density was between 70 and 80% confluent. The culture medium was replaced with medium without serum 2 h prior to transfection. The prepared DNA solution, containing either an enhanced green fluorescent protein (EGFP)-C1 plasmid (40 *µ*l) or a pEGFP-C1-Nlp plasmid (100 *µ*l; Beijing Dingguo Changsheng Biotechnology Co., Ltd., Beijing, China) was added to the tubes and mixed with Opti-MEM medium to a final volume of 2.5 ml (Gibco Life Technologies, Carlsbad, CA, USA). Following addition of the transfection reagent, Attractene (Qiagen, Hilden, Germany) and agitating lightly, Opti-MEM medium was added for 20 min at room temperature. The transfection mixture was transferred into medium containing 5×10^5^ MCF-7 cells and cultured for 8 h in a humidified incubator at 37°C under 5% CO_2_. Following incubation, the medium was replaced with 25 ml fresh 10% serum containing medium for 48 h in a humidified incubator at 37°C under 5% CO_2_. The MCF-7 supernatant was centrifuged at 4,000 x g for 10 min at 4°C and transferred to a filter cup (Sartorius, Goettingen, Germany). The filter cup was then inserted into a filtrate collection tube (Sartorius). The lentivirus was centrifuged at 1,000 x g for 2 min at 4°C and stored at −80°C.

### mRNA expression of Nlp was detected by reverse transcription-quantitative polymerase chain reaction (RT-qPCR)

The total RNA was extracted using TRIzol reagent (Invitrogen Life Technologies, Carlsbad, CA, USA), according to the manufacturer’s instructions. The purity and concentration of the RNA samples were determined using an ultraviolet spectrophotometer (DU 800; Beckman Coulter, Brea, CA, USA) and samples with an absorbance value >1.7 were assessed by RT-qPCR. A total of 1 *µ*g RNA was used to produce cDNA according to the manufacturer’s instructions of the TUREscript 1st Strand cDNA Synthesis kit (Aidlab Biotechnologies Co., Ltd., Beijing, China). The cDNA reaction system was as follows: 1 *µ*l of Oligo(dt)18 (0.5 *µ*g/*µ*l), 10 *µ*l of 2X RT Reaction mix, 1 *µ*l of TUREscript H-RTase/RI mix and RNase free H_2_O at a final volume of 20 *µ*l. The RT-qPCR assays were performed according to the manufacturer’s instructions of the 2X SYBR Green qPCR kit (Aidlab Biotechnologies Co., Ltd., Beijing, China). PCR was conducted using an Applied Biosystems ABI Prism 7000 Real-Time PCR System (Applied Biosystems, Foster city, CA, USA). The cycling conditions were as follows: 94°C for 3 min to activate the DNA polymerase, followed by 40 cycles of 95°C for 40 sec, 61°C for 60 sec and 72°C for 40 sec, and then extended at 72°C for 10 min. The specificity of the amplification products were confirmed by melting curve analysis. The PCR reactions for each gene were repeated three times and independent experiments were performed in triplicate. The primer (Shanghai Jingmei Bioengineering Co., Ltd., Shanghai, China) sequences were as follows: Nlp, forward 5′-ACCTGGGATTCTGAGGACTTTG-3′ and reverse 5′-ACTTTGCCGTCTCCGTCTTGAT-3′ and GAPDH, forward 5′-CATCAAGAAGGTGGTGAAGC-3′ and reverse 5′-GGAAATTGTGAGGGAGATGC-3′. GAPDH was used as an internal loading control.

### Western blotting to detect the protein expression of Nlp and CXCR4

The cells were washed with phosphate-buffered saline (PBS) twice and lysed in radioimmunoprecipitation buffer (50 mM Tris, pH 7.4; 0.15 M NaCl; 1% Triton X-100; 1% sodium deoxycholate; 0.1% SDS) (Aidlab Biotechnologies Co., Ltd.) for 30 min on ice. The samples were then centrifuged at 12,000 x g for 3 min at 4°C and the supernatants were collected and stored at −80°C. Protein concentrations were determined using a bicinchoninic acid assay (Aidlab Biotechnologies Co., Ltd.). The total protein (100 *µ*g) was resolved using a 10% SDS-PAGE gel (Beyotime Institute of Biotechnology), electrotransferred onto polyvinylidene fluoride membranes (GE Healthcare Life Sciences, Little Chalfont, UK) and blocked with 5% non-fat dry milk (Shanghai Bright Dairy Group Co., Ltd., Shanghai, China) for 1 h in Tris-buffered saline (Boster Biological Technology, Ltd., Wuhan, China). The membranes were incubated in primary Nlp polyclonal antibody (1:1,000; cat. no. ab179678; Abcam, Cambridge, CA, USA), rabbit polyclonal C-X-C chemokine receptor (CXCR)4 (1:2,000; cat. no. ab2074; Abcam) and rabbit polycloanl actin antibody (1:5,000; cat. no. sc-7210; Santa Cruz Biotechnology, Inc., Dallas, TX, USA) overnight at 4°C. The membranes were washed with PBS, containing 5% non-fat milk and 0.1% Tween-20, and were subsequently incubated with horseradish peroxidase-conjugated goat anti-rabbit immunoglobulin G antibody (1:2,000; cat. no. SN134; SunShine Bio, Nanjing, China) for 1 h at room temperature. Finally, the membranes were incubated with enhanced chemiluminescence reagents (EMD Millipore, Billerica, MA, USA) for 1 min at room temperature, and developed in a dark room.

### MTT assay to detect cell growth curves

The cells were seeded into 96-well plates (1,000 cells/well) and six wells were repeated. The cells were incubated for 24 h and subsequently treated with 20 *µ*l 5 g/l MTT and incubated at 37°C for 4 h. The supernatants were washed and 150 *µ*l dimethylsulfoxide (Sigma-Aldrich, St. Louis, MO, USA) was added to each well to terminate the reaction. The optical density (OD) value was detected at a wavelength of 570 nm on an enzyme-linked immune detector (Model 680; Bio-Rad Laboratories, Inc., Hercules, CA, USA). The OD value was continuously measured for 6 days, and the growth curves were produced. The day was set as abscissa and the absorbance value as the longitudinal coordinates.

### Plate colony formation assay

The cells were collected as a single cell suspension and were seeded into 6-well plates, each group had three repeated wells and contained 150–200 cells/well. The single cell suspension was incubated in a humidified incubator at 37°C under 5% CO_2_ for 12 days, until the cell colonies were visible by eye. The cells were washed twice with PBS and fixed in 100% methanol (Shenyang Chemical Co., Ltd., Shenyang, China) for 30 min at room temperature. Following fixation, the cells were stained with 0.5% crystal violet (Beyotime Institute of Biotechnology) for 5 min, prior to washing and drying. Positive colonies, containing >50 cells, were observed under a microscope (IX71; Olympus Corporation, Tokyo, Japan), images were captured and colony numbers were quantified.

### Transwell chamber migration assay

For the cell migration assay, 3×10^5^ cells in 1 ml medium without fetal calf serum (FCS) were seeded onto a fibronectin coated polycarbonate membrane insert in a Transwell apparatus (Corning, Inc., Corning, NY, USA). In the lower chamber, 600 *µ*l DMEM, containing 20% FCS was added as chemoattractant. The cells were incubated for 24 h at 37°C in a 5% CO_2_ atmosphere, the insert was washed with PBS and the cells on the top surface of the insert were removed using a cotton swab. The cells adhering to the lower surface were fixed with methanol for 20 min, stained with 5% Giemsa solution (Merck & Co., Inc., Rahway, NJ, USA) for 20 min at room temperature, and quantified under a microscope (IX71; Olympus Corporation) in five randomly selected fields.

### Statistical analysis

Statistical analyses were performed using SPSS 17.0 software (SPSS, Inc., Chicago, IL, USA) and all data are expressed as the mean ± standard error of the mean. A least significant difference-t test was used for two sample comparisons from two groups. P<0.05 was considered to indicate a statistically significant difference.

## Results

### Overexpression of Nlp is established in the MCF-7 breast cancer cell line

**Figure 1:**
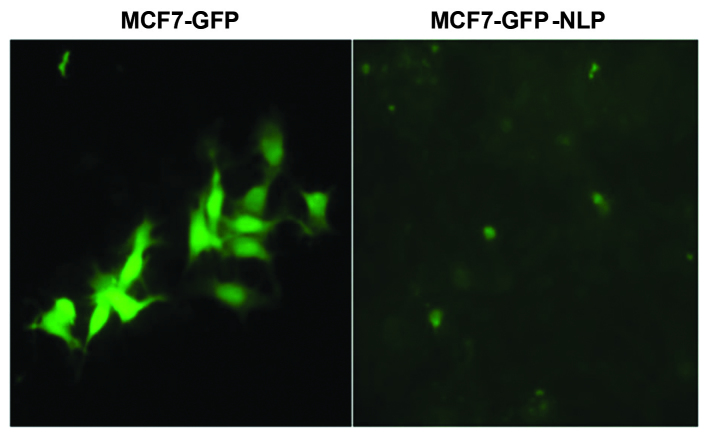
Visualization of the effect of transfection under a fluorescent microscope. When MCF-7 breast cancer cells were transfected with NLP, the transfection efficiency reached 90%, as determined by measuring green fluorescence. (Magnification, x200). GFP, green fluorescent protein; NLP, ninein-like protein.

**Figure 2:**
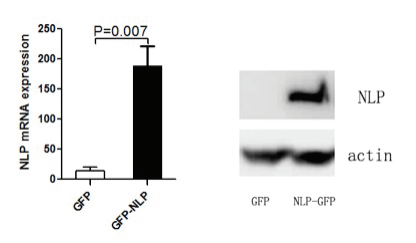
mRNA and protein expression levels of Nlp in the MCF7-GFP-NLP are significantly increased following transfection. The data are expressed as the mean ± standard error of the mean. GFP, green fluorescent protein; NLP, ninein-like protein.

The pEGFP-C1-Nlp plasmid or the pEGFP-C1 plasmid were transfected into MCF-7 cells to establish MCF7-GFP-NLP and MCF7-GFP cells. The mRNA and protein expression levels of Nlp were detected by RT-qPCR and western blotting (Figs. 1 and 2).

### Effect of high expression of Nlp on the growth of MCF-7 cells detected using an MTT assay

**Figure 3:**
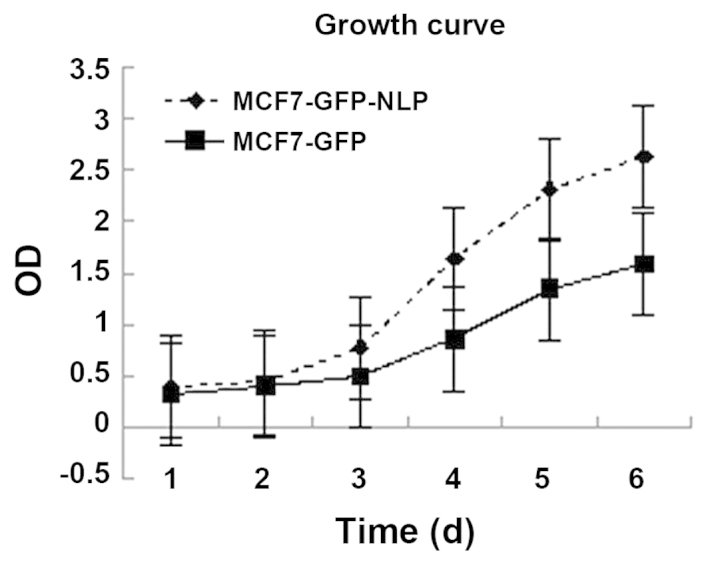
Increased expression levels of Nlp promotes MCF-7 growth (P=0.036). The data are expressed as the mean ± standard error of the mean. GFP, green fluorescent protein; Nlp, ninein-like protein; OD, optical density; d, days.

Under the same growth conditions, the growth rate was more rapid in the MCF7-GFP-NLP cells (P<0.05) compared with the MCF7-GFP cells, which indicated that Nlp promoted the growth of MCF-7 cells (Fig. 3).

### Colony formation assay to detect cell proliferation ability

**Figure 4:**
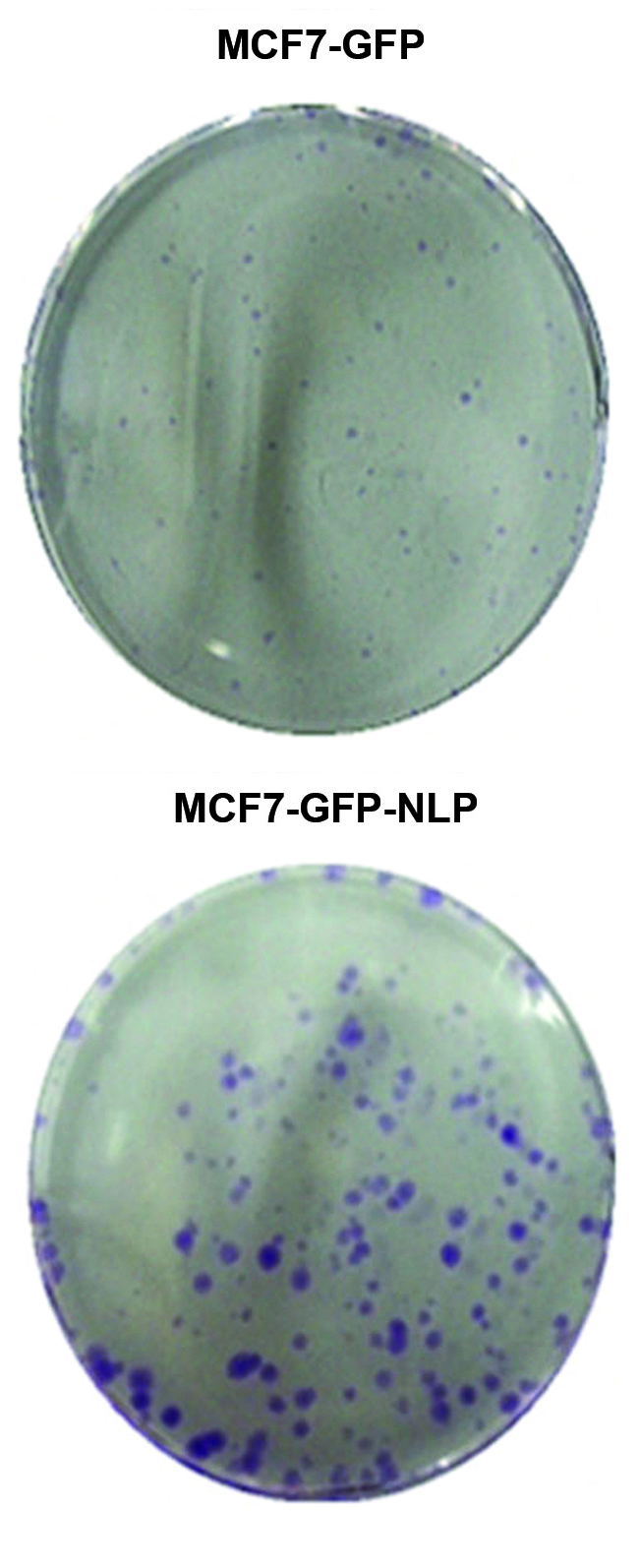
Colony formation in MCF7-GFP cells and MCF7-GFP-NLP cells. GFP, green fluorescent protein; NLP, ninein-like protein.

**Figure 5:**
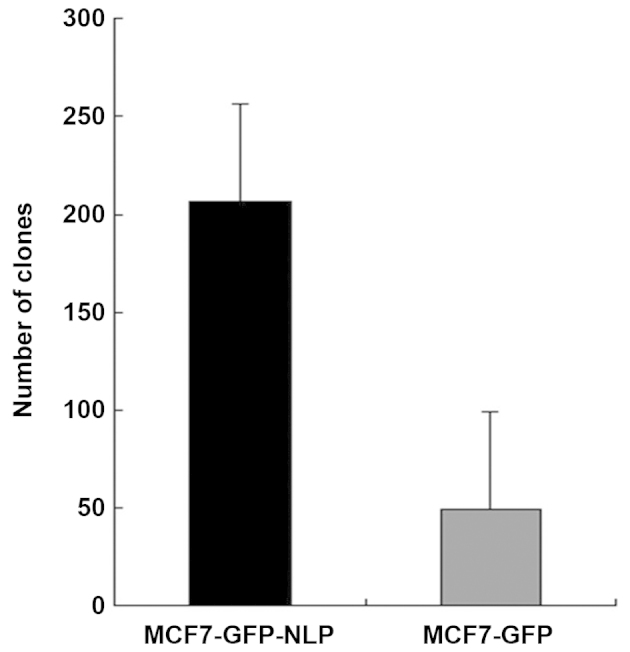
Colony numbers are increased in MCF7-GFP-NLP cells compared with MCF7-GFP cells (P<0.05). The data are expressed as the mean ± standard error of the mean. GFP, green fluorescent protein; NLP, ninein-like protein.

The results demonstrated that colony numbers in MCF-GFP cells and MCF7-GFP-NLP cells were 49±3.45 and 206±14.35, respectively, under identical conditions. The colony formation rate was markedly increased in the MCF7-GFP-NLP cells (P<0.05) compared with the MCF7-GFP cells, which indicated that Nlp promoted MCF7 cell proliferation (Figs. 4 and 5).

### Effect of high expression of Nlp on the migration ability in vitro

**Figure 6:**
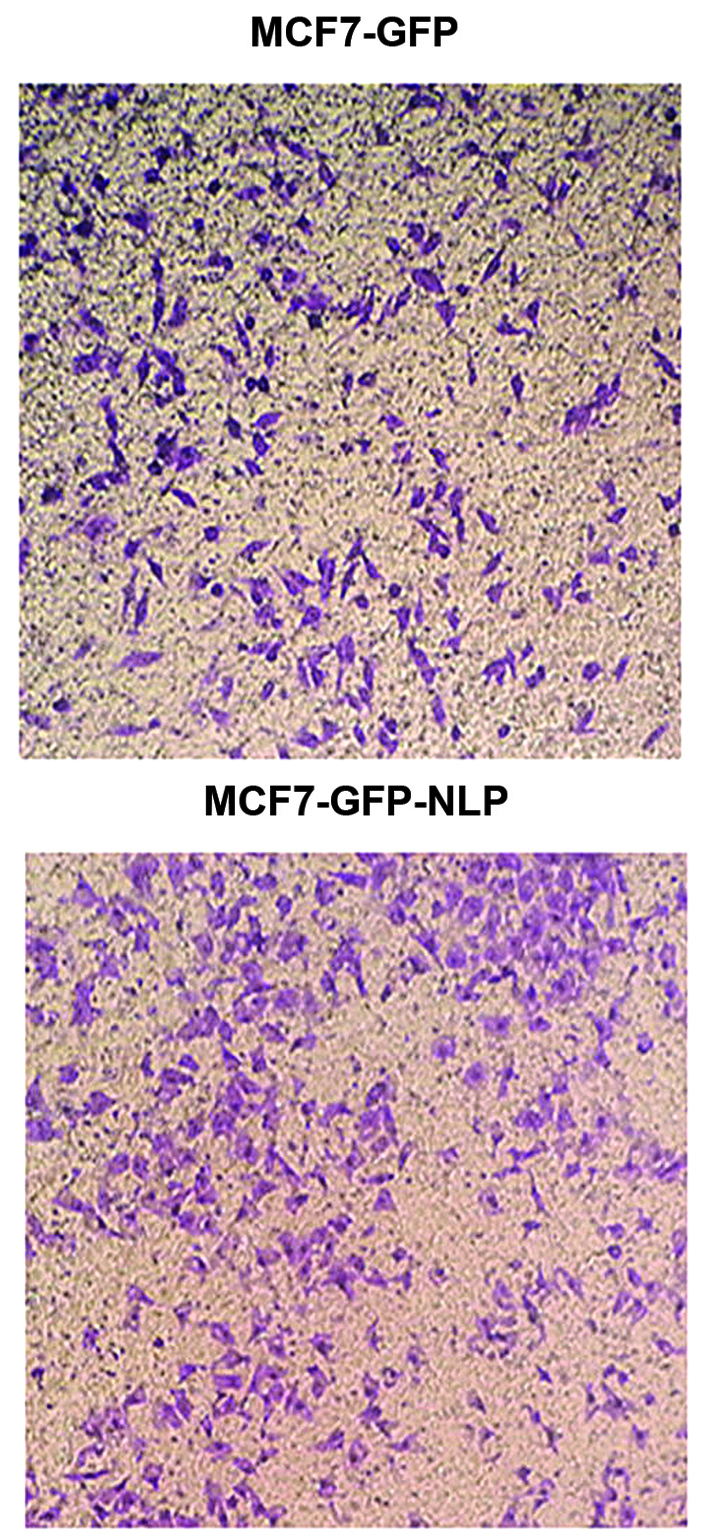
Levels of invasion, observed in a Transwell chamber, between the MCF7-GFP cells and MCF7-GFP-NLP cells. The migratory ability was increased in the MCF7-GFP-NLP cells, as compared with the MCF7-GFP cells. (Magnification, x100). GFP, green fluorescent protein; NLP, ninein-like protein.

**Figure 7:**
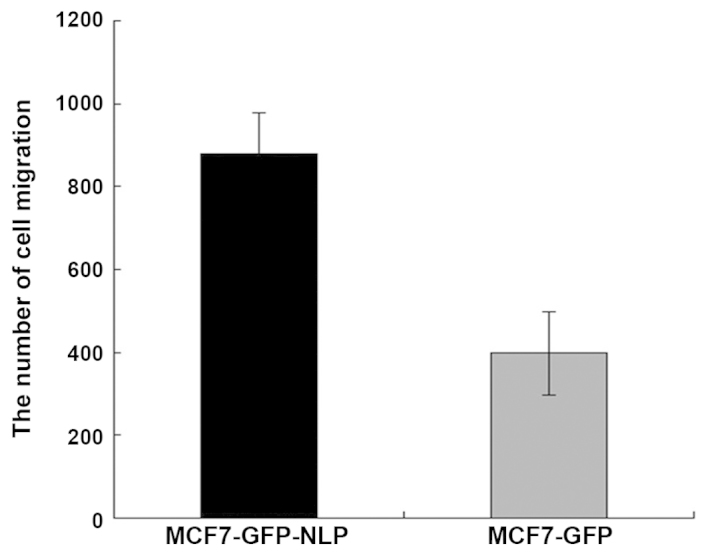
Increased expression of Nlp promotes the migration ability of MCF-7 cells (P<0.05). The data are expressed as the mean ± standard error of the mean. GFP, green fluorescent protein; NLP, ninein-like protein.

Under the same conditions, quantification of the cells migrated under the membrane in MCF7-GFP-Nlp cells and MCF7-GFP cells were 878±18.22 and 398±8.02, respectively. The migration ability was increased in the MCF7-GFP-Nlp cells compared with the MCF7-GFP cells and a significant difference was observed between two groups (P<0.05; Figs. 6 and 7).

### Expression of CXCR4 was detected by western blotting in the MCF7-GFP-Nlp cells and MCF7-GFP cells

**Figure 8:**
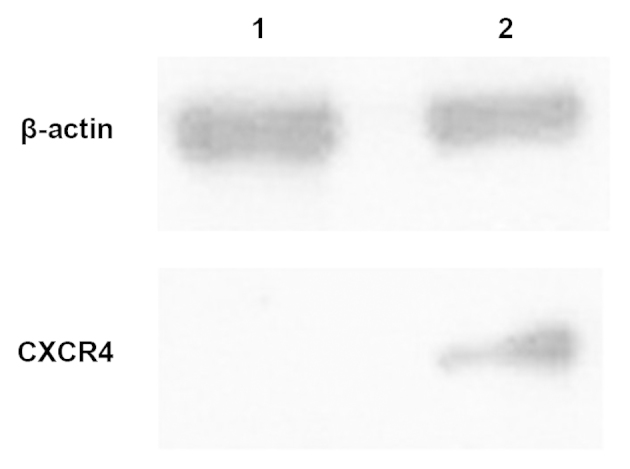
Detection of the expression of CXCR4 in (1) MCF7-GFP and (2) MCF7-GFP-Nlp cells, detected by western blot analysis. GFP, green fluorescent protein; NLP, ninein-like protein; CXCR, C-X-C chemokine receptor.

The results of the western blotting revealed that the protein expression levels of CXCR4 were higher in the MCF7-GFP-Nlp cells compared with the MCF7-GFP cells (Fig. 8).

## Discussion

Nlp is overexpressed in breast, lung and ovarian cancer, and head and neck squamous cell carcinoma. Notably, centrosomal Nlp causes spontaneous tumorigenesis in transgenic mice overexpressing Nlp (10–12). Previous studies have reported that centrosomal abnormalities occur in certain low-grade tumors and exhibit an demonstrate an increased trend in invasive tumors. In ovarian cancer tissues, the higher the pathological classification, the higher the number of centrosome abnormalities that are present. Notably, the presence of centrosome abnormalities are higher in malignant ovarian cancer (13–16). A previous study demonstrated that the overexpression of Nlp is observed in head and neck squamous cell carcinoma, which is associated with the clinicopathological characteristics (17). In addition, it has also been confirmed that the expression of Nlp significantly correlates with the tumor grade, and that the overexpression of Nlp is marginally associated with a decrease in overall survival rates (18). Furthermore, Nlp induces tumor development by interfering with the cell cycle, mitosis and cell apoptosis (19,20). However, the effect of Nlp on breast tumor metastasis remains to be elucidated.

The MCF-7 breast cancer cell line retains several characteristics of differentiated mammary epithelium, including the ability to process estradiol via cytoplasmic estrogen receptors and the capability of forming domes. The present study established MCF7-GFP-Nlp and MCF7-GFP cells and observed, through growth curves that the MCF7-GFP-Nlp cells grew more rapidly compared with theMCF7-GFP cells. In addition, plate colony forming assays demonstrated that the MCF7-GFP-Nlp cells exhibited a increased colony formation capacity compared with the MCF7-GFP cells. These results indicated that Nlp promoted MCF-7 cell proliferation. Transwell chambers are considered as a permeability support, and usually, a Transwell chamber is put into culture plates and medium is added to the top and bottom chambers, with the cells were seeded into the top chamber. Since the membrane is permeable, cells can migrate to the lower chamber (21,22). The results of the present study revealed that the overexpression of Nlp promoted cell migration in the Transwell model *in vitro*.

Changes in tumor cell migration capacity is an important step affecting tumor invasion and metastasis. CXCR4 is encoded by 352 amino acids and is a seven-transmembrane G-protein chemokine receptor. In 1996, Feng *et al* identified that CXCR4 is a coreceptor for human immunodeficiency virus-1 entry, following which several studies have investigated CXCR4 (23). It has been demonstrated that CXCR4 is involved in the invasion and metastasis of several types of cancer, including breast carcinoma (24). Hiller and Chu (25,26) demonstrated that CXCR4 is important in several types of cancer, including breast cancer, and revealed that CXCR4 was highly expressed in areas common for breast cancer metastasis, including the axillary lymph nodes. Hernandez *et al* confirmed that CXCL12-CXCR4 is important in the process of breast tumor cell growth, angiogenesis, invasion and metastasis (27,28). A meta-analysis investigation based on thirteen eligible studies, consisting of 3,865 patients with breast cancer, demonstrated that the overexpression of CXCR4 was significantly associated with lymph node status and distant metastasis. In addition, the overexpression of CXCR4 indicated a poor overall and disease-free survival rates (29). The present study demonstrated that the expression of CXCR4 was higher in the MCF7-GFP-NLP cells compared with the MCF7-GFP control cells, which implied that Nlp improved the migration capacity of breast cancer cell lines through activated CXCL12 and CXCR4.

In conclusion, the results of the present study indicated that an increase in the expression of Nlp resulted in a malignant phenotype, which induced tumor cell proliferation and invasion. Furthermore, the results confirmed that Nlp exhibited certain biological characteristics, including promoting breast tumorigenesis and development, to provide a novel molecular index for breast cancer diagnosis. Therefore, Nlp may be an effective target of antitumor drugs for therapy against specific types of tumor.
